# Opto-Mechatronics System for Train-Track Micro Deformation Sensing

**DOI:** 10.3390/s22010296

**Published:** 2021-12-31

**Authors:** Weibing Gan, Shiyu Tu, Yuan Tao, Lingyun Ai, Cui Zhang, Jianguan Tang

**Affiliations:** 1 National Engineering Laboratory for Fiber Optic Sensing Technology, Wuhan University of Technology, Wuhan 430070, China; ganweibing@whut.edu.cn (W.G.); taoyuan0801@163.com (Y.T.); 10288761@zte.com.cn (L.A.); tangjg@whut.edu.cn (J.T.); 2 School of Information Engineering, Wuhan University of Technology, Wuhan 430070, China; yogaliving@163.com

**Keywords:** low-reflectivity fiber Bragg grating array, WDM/TDM hybrid networking, opto-mechatronics technology, train-track system, micro deformation

## Abstract

In this paper, we proposed and experimentally demonstrated an opto-mechatronics system to detect the micro-deformation of tracks caused by running trains. The fiber Bragg grating (FBG) array acting as sensing elements has a low peak reflectivity of around −40 dB. The center wavelengths were designed to alternate between 1551 nm and 1553 nm at 25 °C. Based on dual-wavelength, wavelength-division multiplexing (WDM)/time-division multiplexing (TDM) hybrid networking, we adopted optical time-domain reflectometry (OTDR) technology and a wavelength-scanning interrogation method to achieve FBG array signal demodulation. The field experimental results showed that the average wavelength shift of the FBG array caused by the passage of the lightest rail vehicle was −225 pm. Characteristics of the train-track system, such as track occupancy, train length, number of wheels, train speed, direction, and loading can be accurately obtained in real time. This opto-mechatronics system can meet the requirements of 600 mm spatial resolution, long distance, and large capacity for monitoring the train-track system. This method exhibits great potential for applications in large-scale train-track monitoring, which is meaningful for the safe operation of rail transport.

## 1. Introduction

The rail transport industry plays a vital role in many countries’ infrastructures. While producing huge economic benefits, the safe operation of the rail system is a critical issue. Serious rail traffic accidents occur frequently around the world every year. Many accidents in rail transport are caused by faults in the train and track systems [1]. How to safely, efficiently, and accurately monitor the state of train tracks is a significant problem that needs to be solved.

At present, well-developed methods for checking track status include track circuits [2], image recognition [3,4], and the global positioning system (GPS) [5,6]. The common feature of these technologies is that they are all based on electronic technologies, which are vulnerable to interference from electromagnetic fields, the external environment, and weather conditions.

Compared with electrical sensing technology, optical-fiber sensing technology has many advantages, such as proof of electromagnetic interference, corrosion resistance, their light weight and small size, low transmission loss, high precision, and fast response, as well as the fact that they are easy to network [7,8].

The fiber Bragg grating (FBG) sensor is a mainstay of fiberoptic sensing technology. Since K.O. Hill [9] fabricated Bragg gratings in germanium-doped fiber with an argon-ion laser, there are many fabrication methods for FBG. It was not until 1989 that Meltz, an American scholar, and his colleagues successfully made FBG using the side-exposure method with high-intensity ultraviolet (UV) light, after which grating production efficiency was greatly improved, laying a technical foundation for engineering mass production [10]. After decades of gradual development, the mass-production technology of FBG has become quite mature, and the production cost has been greatly reduced, which has greatly promoted the rapid development of FBG sensors in various industries [11,12,13,14,15].

The unique advantages of FBG sensing technology make it suitable for applications in the rail transport industry. In the past decade, much research progress has been achieved for applications of FBG technology [16,17,18,19,20,21]. In 2010, Wei, C. L. et al. proposed a shaft-counting device based on optical fiber sensing technology, which can detect the number of wheel axles passing overhead [22]. In 2012, Filograno, M. L. et al. installed four FBG sensors on the operating track and conducted testing experiments on different types of trains. The experimental results showed that the FBG sensors achieved good results in train recognition, axle-counting, and speed and acceleration detection [23]. In 2017, Kyle Arakaki et al. studied acoustic emissions generated by train-track systems, using FBG and high-resolution, low-cost optical readers developed by PARC [24]. In 2019, Zhang, S.L. et al. designed a track-circuit monitoring system based on optical fiber sensors. The feasibility of their system in terms of track monitoring was proved [25]. In 2019, Zhang, C. et al., from the Wuhan University of Technology, obtained the force distribution of the track between adjacent sleepers through a finite element analysis of the train-track system. The structure and fabrication process of the low-reflectivity FBG sensor were designed. Calibration experiments showed that the FBG sensor offered good linearity and repeatability [26].

Although FBG sensing technology has many advantages over electrical sensing technologies, it also faces a fundamental scientific limitation. The multiplexing capacity of the FBG sensor is limited by the bandwidth of the laser source and FBG. For a light source with a 40 nm bandwidth, for the sake of safety, each optical channel can only access up to 20 FBGs in engineering applications [27]. As a result, it cannot be used for large-scale train-track status detection.

In view of the technical bottleneck of FBG in long-distance detection, there is an urgent need to develop a long-distance, high-reliability, high-accuracy and weather-resistant train-track occupancy recognition monitoring system. This system should be able to provide continuous and reliable information to the train positioning system and train control system, which helps to maintain the appropriate spacing between trains and guarantees the safe operation of train networks.

In this paper, an opto-mechatronics train-track status monitoring system based on a low-reflectivity FBG array is designed. The FBG array we prepared has a low reflectivity (−40 dB) and was inscribed alternately with two wavelengths (1551 nm and 1553 nm, based on an environmental temperature of 25 °C). Tens of thousands of low-reflectivity FBGs can be arranged in series on a single fiberoptic cable. A dual-wavelength FBG array wavelength-division multiplexing (WDM)/time-division multiplexing (TDM) hybrid multiplexing system has been established. Optical time-domain reflectometry (OTDR) technology is used to interpret each FBG signal correctly. In practice, the FBG array was installed on the ballast track to sense the micro-deformation of the track when the train passed by. By analyzing the micro-deformation of the track, not only the track occupation information can be determined but also the weight, the speed, the direction and the information of the wheel and axle can be obtained simultaneously. Different from single WDM and TDM technology, due to the narrow bandwidth and low reflection of the FBG array, the limitation of the spectral width of the light source and transmission loss is greatly reduced. The multiplexing capacity and sensing distance are greatly increased accordingly. The technology offers a promising solution for long-distance, high-capacity train-track occupancy recognition and online monitoring.

## 2. Optical System: The Low-Reflectivity FBG Array and Its Multiplexing Network

### 2.1. Low-Reflectivity FBG Preparation and Sensing Principle

**Figure 1:**
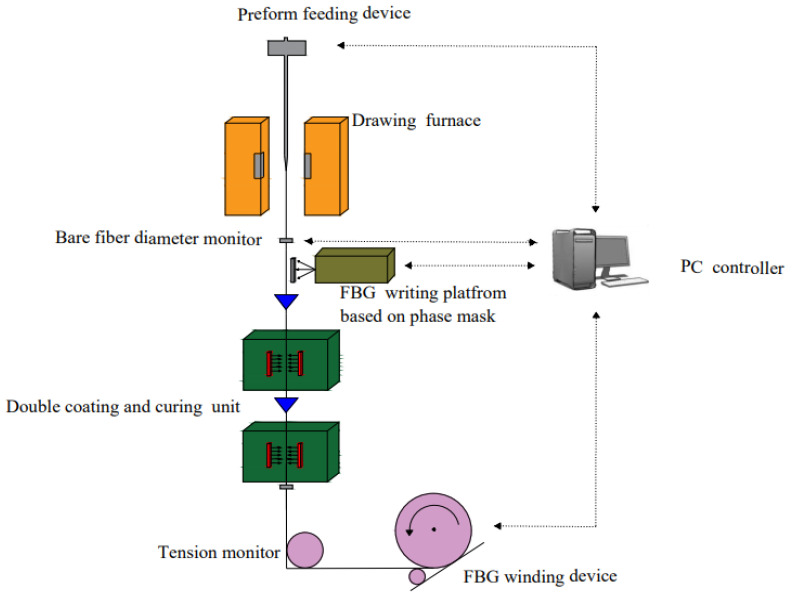
Schematic diagram of the FBG array drawing tower in-line fabrication system.

A low-reflectivity FBG is also known as a draw-tower grating (DTG) [28]. The grating is written synchronously into low-loss, single-mode fibers in the process of optical fiber drawing, based on our customized drawing-tower in-line fabrication system. The drawing and writing equipment, as shown in Figure 1, includes a high-temperature graphite resistance furnace, fiber diameter monitor, phase mask, UV excimer laser, coating unit, winding device, tension monitor, computer, and so on. Low-reflectivity FBGs are formed by the periodic modulation of the refractive index on the core of a single-mode fiber. The spacing between two adjacent FBGs can be precisely controlled under a stable drawing speed. The fabricated FBGs have a defect-free coating layer and no splicing joint, ensuring greater mechanical strength [29]. The low-reflectivity FBG is a uniform periodic grating and reflects the light at one particular wavelength called the “Bragg wavelength” only when it satisfies the Bragg condition. Its sensing principle follows the mode-coupling theory of FBG [30]. The Bragg wavelength of FBG is modulated simultaneously by temperature and strain. The relationship between the wavelength shift with temperature and strain can be explained by Equation (1) [15]:

(1) $$\frac{\Delta \lambda_{B}}{\lambda_{B}}=(1-P_{e})\Delta \varepsilon +(\alpha +\zeta_{f})\Delta T$$

where *λ_B_* is the Bragg wavelength, Δ*λ_B_* is the change of Bragg wavelength, *P_e_* ≈ 0.22 is the photoelastic component, *α* is the thermal optical coefficient of the fiber, *ζ_f_* is the thermal expansion coefficient, Δ*ε* is the change of strain, and Δ*T* is the change of temperature. When the FBG is only affected by axial stress, the elastic–optic effect of the optical fiber changes the FBG period and the effective refractive index and results in an FBG wavelength shift. According to Formula (1), assuming that the ambient temperature around the FBG is constant, the relationship between strain and wavelength can be described as:

(2) $$\frac{\Delta \lambda_{B}}{\lambda_{B}}=(1-P_{e})\Delta \varepsilon =K_{\varepsilon }\Delta \varepsilon$$

where *K_ε_* is the strain sensitivity of the FBG; then, the FBG wavelength shift caused by strain can be expressed as:

(3) $$\frac{\Delta \lambda_{B}}{\Delta \varepsilon }\approx 0.78\lambda_{B}$$

If the FBG has a Bragg wavelength of 1551 nm, its wavelength shift, caused by the unit strain, is 1.209 pm.

It can be seen from the above FBG sensing principle that the FBG wavelength is demodulated simultaneously by external temperature and strain. In practical engineering applications, a temperature influence filter is needed to ensure the accuracy of detection. So, for track-strain measurement, the cross-sensitivity of temperature and strain must also be considered.

There are many ways to decouple temperature and strain. The easiest and most common method is by using the FBG temperature-compensation method. In the temperature-compensation method, one FBG temperature sensor is installed in the same temperature field as the FBG strain sensor. The temperature response of the strain sensor is compensated for by the temperature sensor.

In this system, the FBG sensor senses not only the micro-deformation of the track caused by a passing train but also the ambient temperature variation; the change of temperature is relatively slow compared with the track strain rate. Thus, the temperature signal can be regarded as a direct-current (DC) signal. In the software algorithm of this system, a digital filtering method has been adopted to filter out the temperature effect without adding an additional FBG sensor for temperature compensation. In addition, the influence of ambient temperature on strain monitoring is further reduced by the method of online automatic zero-point correction. According to the real-time status of the train-track system, the system software will analyze the wavelength variation trend of each FBG sensor every 24 h or over several hours (user-configurable) and correct the reference base value when the track is in the idle state. This method avoids the temperature error when the zero point is fixed. These two methods can effectively reduce the influence of temperature on the train-track monitoring system.

### 2.2. Dual-Wavelength Low-Reflectivity FBG WDM/TDM Hybrid Multiplexing Network for Train-Track Monitoring System

Multiplexing networking modes of FBGs mainly include WDM, TDM, space division multiplexing (SDM), and mixed multiplexing. Here, this mainly refers to the FBG hybrid multiplexing mode based on WDM and TDM.

The WDM technology of the FBG means that different wavelength optical signals are multiplexed into one optical fiber for transmission. When an incident light signal travels through the fiber and reaches one FBG, the same wavelength as the FBG’s Bragg wavelength is reflected. Different gratings have different Bragg central wavelengths, and the reflected spectrum signals also vary accordingly. The position of the FBG can be distinguished and recognized using different central wavelength information. In actual applications, WDM requires that the dynamic operating range between FBG sensors be set to ±1 nm up to ±2 nm (or more than 2 nm in special cases) in order to prevent signal crosstalk. However, the bandwidth of the common light source is about 40–80 nm. The dynamic range required by the measured variable and the limited bandwidth of the light source limit the amount of wavelength-division multiplexing of the sensor, which seriously restricts the application of FBGs in conditions requiring long distances and large capacity.

TDM interweaves different signals at different time periods and transmits them along the same channel. Due to the differences in the optical path, the optical signal that is reflected back carries the position information of the FBG. In TDM systems, the number of FBGs multiplexing is independent of the central wavelength of the FBG and the bandwidth of the system. The time of receiving the FBG reflection signal can be expressed by Equation (4):

(4) $$\tau_{i}=\frac{2n_{eff}L_{i}}{c}$$

where *τ_i_* is the time of receiving FBG signal, *L_i_* is the length from the light source to the FBG, *n_eff_* (≈1.4682 for SMF-28 fiber at 1550 nm) is the effective refractive index of the fiber core, and *c* (≈3 × 10^8^ m/s) is the propagation speed of light in a vacuum.

The modulated light pulse signal has a certain width. If the transmission time of the optical signal between two adjacent FBGs is less than the width of the optical pulse signal, then the detector cannot accurately distinguish the optical signals reflected by the two FBG, which is called signal crosstalk and can easily cause signal demodulation errors and other problems. Therefore, in this system, the distance between two adjacent FBGs must meet the condition that the round-trip transmission time of the optical pulse signal is greater than the pulse width.

In TDM systems, the spatial resolution of the sensing system is limited by the existing photoelectric devices, especially the pulse width of a tunable laser. Based on OTDR technology, a 20 nm pulse width of a laser can obtain a spatial resolution of 2 m. For the experimental ballast track, the spacing between two adjacent sleepers is generally about 600 mm. The redundant optical fiber between the two sleepers in a 2-m spatial resolution TDM system will be too long, which makes it prone to damage by rail traffic and external disturbances. According to a previous finite-element analysis of a track-train system [26], the deformation is greatest in the middle of the track between two sleepers. If one FBG is placed between every two sleepers, a spacing of 1 m between each sensor is most appropriate. Therefore, a single TDM system cannot meet the needs of the system. This spatial resolution needs to be further improved.

**Figure 2:**
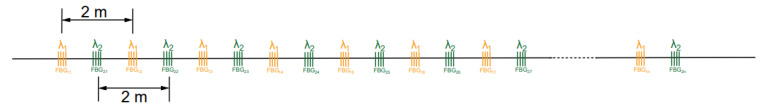
The schematic diagram of a dual-wavelength low-reflectivity FBG array.

A WDM/TDM hybrid multiplexing network can solve the above two problems simultaneously. In this paper, we present a dual-wavelength WDM/TDM hybrid multiplexing network, based on a low-reflectivity FBG array. The schematic diagram is shown in Figure 2.

The FBG array, acting as a sensing element, can be regarded as a series of low-reflectivity FBGs arranged continuously at certain spatial intervals. It has low reflectivity (−40 dB) and is inscribed alternately with two wavelengths (λ_1_ = 1551 nm and λ_2_ = 1553 nm). The spacing of λ_1_ and λ_2_ is 1 m and the same-wavelength FBGs are spaced 2 m apart. The multiplexed number of FBG sensors with identical wavelengths can be set according to the need for a particular detection length of rail (here, this is 100 FBGs per wavelength). This dual-wavelength WDM/TDM quasi-distributed low-reflectivity FBG array network can meet the requirements of long distance, large capacity and high-resolution train-track status monitoring.

Through the WDM/TDM hybrid multiplexing mode, the problem of FBG signal cross-talking can be solved to a large extent; likewise, the spatial resolution can be doubled without increasing the cost and the complexity of the demodulation system. This demodulation principle will be described in detail in the fourth part of this paper.

## 3. Mechanical Systems: Train-Track Force Analysis and Sensor Encapsulation Size

Due to the particularity of railway transit, drilling, welding, and any series of operations to destroy the track structure are not allowed on the track. The only method currently available is to attach a sensor unit to the track with special fixtures. Zhang’s previous finite element analysis of the train-track system leads to the following conclusions: when the load is directly over the sleeper, track deformation is smallest, and stress is mainly concentrated at the track head; when the load is applied right between two sleepers, the track deformation and stress are the greatest, and the maximum deformation of the track is at the track head and waist [26]. Therefore, an FBG sensor should be installed between two sleepers and near the rail waist and railhead for maximum strain-sensing capability.

When installed, each FBG sensor is fastened to the side of the track by two clamps, and a certain amount of pre-tension is applied to FBG by fine-tuning jigs. When the train runs over a certain section of the test track, the weight of the train is applied to the track between two sleepers through the wheels. When the track deforms, the relative position of the two clamps held on the sides of the track changes. This changes the FBG initial pre-tension, then causes its wavelength to shift. At this point, the track strain value can be calculated according to the wavelength variation.

**Figure 3:**
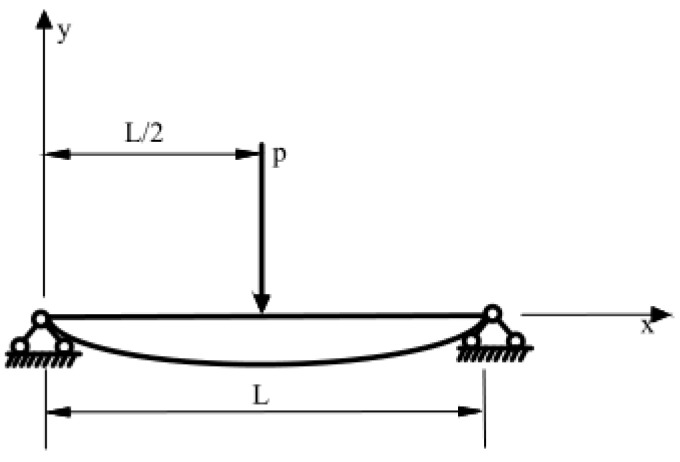
Deformation diagram of a simply supported beam.

FBGs can withstand large wavelength variations, generally up to ±5000 pm. In this practical application of track occupancy monitoring, considering the FBG sensor installation and signal demodulation system, we only need to be able to obtain a track deformation signal with a certain signal-to-noise ratio (SNR), and it is not necessary to blindly increase the strain sensitivity coefficient of the FBG. Due to the limitation of the distance between two track sleepers, it is necessary to further calculate and analyze the most optimal distance scheme between the FBG sensor package length and the fixture. We have taken the ballast track as an example; the distance between adjacent sleepers is 600 mm. The track between two sleepers is taken as the research object, and it is simplified into a simply supported beam for analysis and research. Figure 3 shows the system simplification.

According to the mechanics of materials, its equation of deflection curve can be described as in Equation (5), where $0\leq x\leq \frac{L}{2}$:

(5) $$y=-\frac{Px}{48EI}(3L^{2}-4x^{2})$$

where *P* is the load acting on the simply supported beam, *E* is the elastic modulus of the beam, *I* is the moment of inertia of the beam section, and *L* is the distance between two ties. We take the derivative of Equation (5) to obtain the offset angle formula of point *X*_0_ on the simply supported beam. The offset angle formula is shown in Equation (6):

(6) $$\theta =\arctan\left[\frac{P}{16EI}(4x_{0}^{2}-L^{2})\right]$$

**Figure 4:**
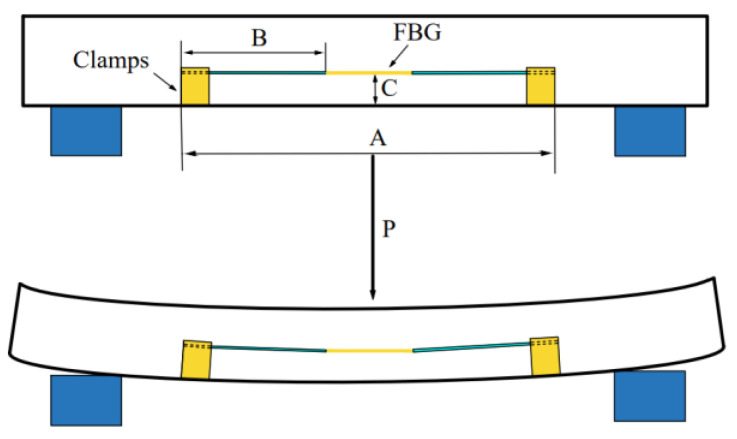
Schematic diagram of ballast track deformation.

When the track is deformed, the relative position of the two fixtures on one side of the track will change, thus changing the pre-tension of the FBG. The deformation diagram is shown in Figure 4.

In Figure 4, *A* is the distance between the two fixtures, *B* is half of the axial length of the FBG sensor, pasted and encapsulated with the external components, *C* is the distance between the FBG and the bottom of the fixture, and *P* is the load.

**Figure 5:**
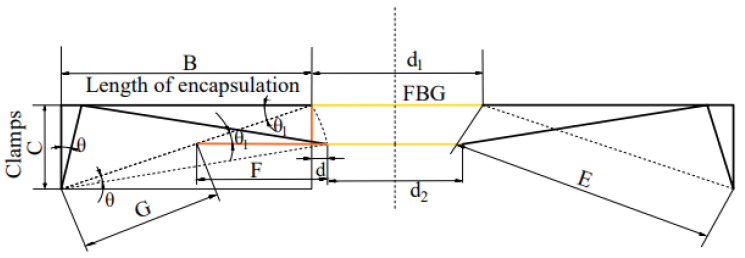
Equivalent schematic diagram of micro-deformation of the installed FBG.

In order to facilitate the analysis of FBG microstrain, the equivalent schematic diagram and auxiliary lines are presented, as shown in Figure 5.

In Figure 5, *d* is one-half of the shortened amount of the optical fiber after the track deformation. The relationship between the shortened length of the optical fiber, *D,* and the axial length of the sensor, *B,* can be obtained through calculation. The relationship can be expressed, as in Equation (7).

(7) D=2d=Bcosθ+Csinθ−B

We substitute Equation (6) into Equation (7) to get Equation (8), as follows:

(8) $$\begin{array}{l} D=B\cos\arctan\left[\frac{P}{16EI}(4x_{0}^{2}-L^{2})\right]\\ +C\sin\arctan\left[\frac{P}{16EI}(4x_{0}^{2}-L^{2})\right]-B \end{array}$$

The experimental conditions are as follows: with 20 MPa load, 8 cm^2^ contact area, then, *P* is 8000 N, with 600 mm sleeper spacing (*L*), and 70 mm distance between the optical fiber and the bottom of the fixture (*C*). The elastic modulus (*E*) of the track is 2.06 × 10^5^ MPa and the cross-section moment of inertia of the track, *I*, is 260 cm^4^. We substitute these parameters into Formula (6) to obtain the formula of fiber shortening, *D,* and sensor length, *B*, as shown in Equation (9):

(9) $$D=-3.808\times 10^{-9}\times B+0.0061$$

The axial strain of the optical fiber is solved according to the linear strain formula, as shown in Equation (10):

(10) $$\varepsilon =\frac{\Delta L}{L_{0}}$$

where Δ*L* is the expansion amount, and *L*_0_ is the length before expansion; then:

(11) L=250−2×B

Then, we substitute Equations (3), (10) and (11) into Equation (9) to obtain the relationship between the sensor length, *B,* and wavelength shift of FBG, as shown in (12).

(12) $$\Delta \lambda_{B}=\frac{-0.0046\times B+7394.89}{250-2\times B}$$

According to the above equation, we choose *B* <= 117.6 mm, so as to ensure that the wavelength variation of FBG (Δ*λ_B_)* is between 0 and 500 pm.

## 4. Electrical System: Dual-Wavelength Low-Reflectivity FBG Array WDM/TDM Hybrid Multiplexing Signal Interrogation

**Figure 6:**
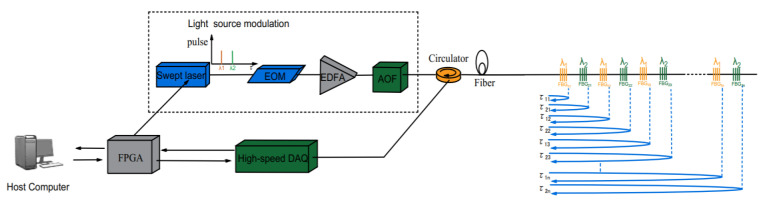
Framework of a dual-wavelength WDM/TDM hybrid multiplexing interrogation system.

According to the above analysis, dual-wavelength WDM/TDM hybrid multiplexing technology can be used to network a low-reflectivity FBG array. OTDR technology and the wavelength-scanning method are used to analyze each FBG signal. The overall framework of this system is shown in Figure 6.

Continuous light from the swept laser source is converted into pulse light after the modulator. After being amplified by erbium-doped fiber amplifiers (EDFA), the pulse signal is transmitted through a fiber-optic circulator to the low-reflectivity FBG array. The other end of the optical circulator selects and isolates the optical pulse and transmits a photosensitive signal to the photoelectric conversion module for photoelectric conversion. The electrical signal after photoelectric conversion enters a high-speed signal-processing element with a field-programmable gate array (FPGA) as the control core. The logic realization of the whole system is based on FPGA. Finally, all of the data is communicated to the host computer. The final signal is processed in the host computer, and a correlative demodulation algorithm is used to demodulate the total information of the FBG array to obtain such parameters as wavelength, return time and position information, and so on.

The specific demodulation algorithm is as follows: computer systems act in each sampling cycle by setting a threshold value to collect valid data. The spectral information of FBG at different positions is intercepted for spectrum splicing. Multi-point moving mean filtering is performed on the sampled spectrum. A Gaussian Levenberg–Marquardt (Gaussian-LM) algorithm is used to fit the spectrum of splicing spectral data at different locations. We calculate the spectral peaks at different locations. Finally, the FBG peak information at different positions is managed by partition, and the wavelength sequence of FBG is output according to the actual physical position.

Because this system is a dual-wavelength WDM/TDM system, its scanning range of light sources in each scanning cycle is set to *λ*_1_ − 500 pm~*λ*_2_ + 500 pm, where *λ*_1_ represents the starting wavelength of the light-source scanning and λ_2_ represents the cutoff wavelength of the light-source scanning. The wavelength scanning step is set to Δ*λ*, then the number of scanning optical pulses *n = (λ*_2_ *− λ*_1_*)/*Δ*λ* + 1. We start the light source to scan the initial input light pulse signal wavelength, λ_1._ The wavelength of the second input optical pulse is *λ*_2_ *= λ*_1_ *+* Δ*λ*. The third is *λ*_2_ *= λ*_1_ *+* 2 × Δ*λ*. Similarly, the wavelength of the Nth input optical pulse is *λ*_2_ *= λ*_1_ *+ (n −* 1*)* × Δ*λ*. When the wavelength of the input optical pulse is *λ_2_* + 500 pm, the system has completed the scanning of one cycle. At this time, collected data are transmitted to the host computer for subsequent analysis, and the optical pulse-scanning task of the next cycle will be started, to make the WDM/TDM system work continuously.

Our low-reflectivity FBG array is composed of two different wavelengths, *λ*_1_ and *λ*_2_. The two central wavelengths are 1551 nm of *λ_1_* and 1553 nm of *λ_2_*, respectively. To ensure that all FBGs can be “addressed” correctly, the swept laser starting wavelength is set to 1550.5 nm, the cutoff wavelength is set to 1553.5 nm, and the scanning step is set to 20 pm.

## 5. Field Trial and Real-Time Feature Analysis of Train-Track Status

Field experiments of this opto-mechatronics system were carried out on one non-operating track in a railway signal plant. Experimental track material is standard 45 steel (density ≈ 7.85 g/cm^3^) to Chinese National Standard (CNS). Since this is a non-operational track, we used an idle trailer on a flatcar, TOFC (the lightest rail vehicle), for confirmative experiments. The TOFC weighs 18 tons and is 13 m long, with a total of four pairs of axles, including the front and rear sets of 8 wheels (single wheel-bearing weight, 2.25 tons). The distance between the first and fourth sets of axles is 11.2 m. A dynamic test was carried out by manual implementation. The length of the test track is about 200 m.

### 5.1. Low-Reflectivity FBG Array Sensor Installation

Rail transport is closely linked to the safety of passengers’ lives and properties. No sensor is allowed to be installed on a rail by pasting, welding, or other methods that may damage the track structure. After discussion and laboratory demonstration, we decided to attach the FBG sensor array to the track by using a novel, dedicated fixture of our own design.

**Figure 7:**
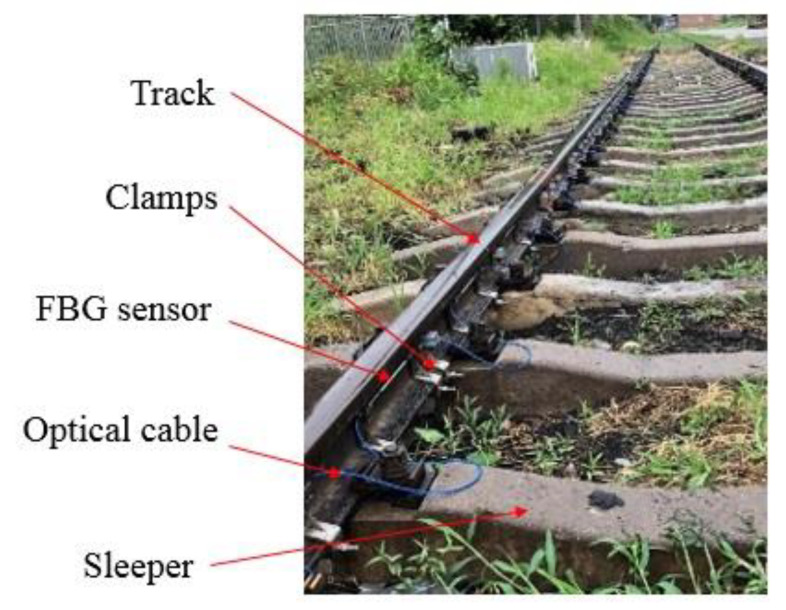
Installation of a low-reflectivity FBG array sensor on the track at the field site.

This installation method will not affect the track’s mechanical properties and can also ensure the safety and reliability of the FBG array. Because one side of the track needed to be installed with other kinds of sensors for additional experiments, our FBG detection system could be only installed on the other side of the track. According to train-track system requirements, one FBG is installed in the middle of the track between two adjacent sleepers. The adjacent sleeper spacing is 600 mm, and the FBG spacing is 1 m. Each FBG is fastened to the inner side of the track by two fixtures. Under real-time wavelength monitoring, 500–1000 pm pre-tension is applied to each FBG by the fine-tuning function of the fixtures. The field installation of the FBG array sensor is shown in Figure 7.

### 5.2. Experimental Results and Discussion

This opto-mechatronics system can follow and locate the track occupancy in real time by analyzing the wavelength shift of the FBG array sensor, caused by the micro-deformation of the track. Based on this, many characteristics of both train and track can also be analyzed and assessed. For example, the number of sensor wavelength peaks can be counted to obtain the wheel axle information of the train. The load of the train itself can be inferred from the FBG absolute wavelength shift and wheel-axle load. Speed and direction can also be established by the interval time between wheels passing two adjacent sensors in turn.

During the field trial, the TOFC entered the test area from the first FBG sensor, and then returned from the last sensor. Because the data of the go-and-return path are quite consistent, we only selected several FBG sensors from when the TOFC entered the detection area for analysis.

#### 5.2.1. Track Occupation Recognition and Location

**Figure 8:**
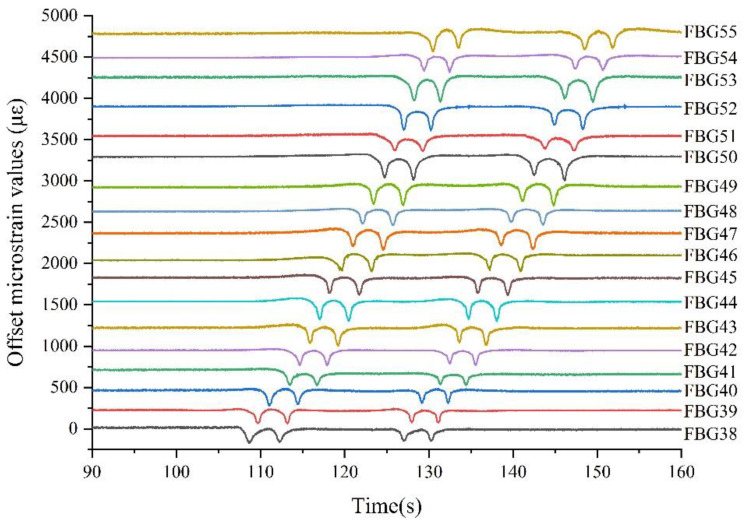
Response of partial FBG sensors when the TOFC is passing.

Figure 8 shows the responses of the partial FBG sensors when we ran the TOFC. The TOFC entered the track detection area from FBG1.

As can be seen from Figure 8, when the TOFC entered the detection area and passed the sensors from the 38th to the 55th, the FBGs responded in turn. Because the FBG arrays were only installed on one side of the tested track, each FBG responded four times. This corresponds exactly to the four axles on one side of the TOFC. As the TOFC continued to move forward, those FBGs corresponding to the physical position of the TOFC responded one by one. The FBG’s wavelength drift reached its maximum (absolute value of wavelength shift) when the wheel was directly above the FBG. The physical location of the FBG position with the largest response corresponds to the TOFC position. Through the responses of every sensor, the TOFC can be located and tracked accurately in real time. Obviously, taking FBG1 as the starting point, the portion of track from 38 m to 55 m was occupied during the period of 108 s to 150 s.

**Figure 9:**
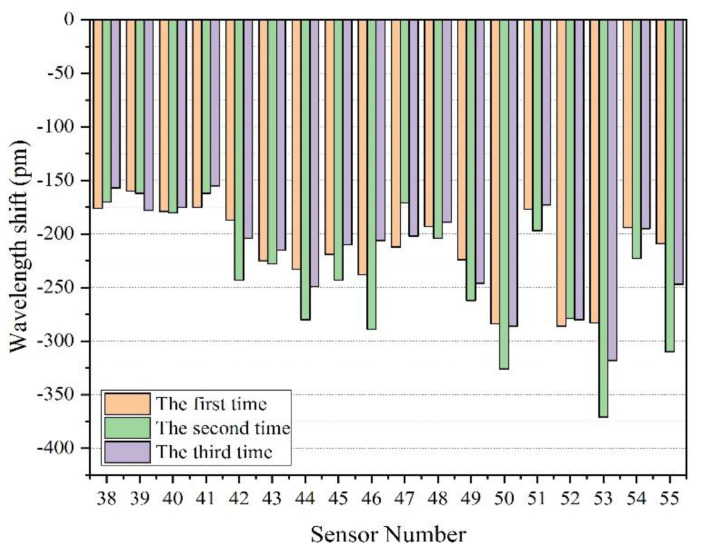
Data statistics of the partial FBG sensor-response peak in three experiments.

In order to verify the repeatability of the measurement, we have performed many driving experiments. We counted certain FBG sensors’ wavelength shifts from three driving experiments; the statistical data is as shown in Figure 9.

As mentioned above, each FBG sensor was equipped with a pre-tension of 500–1000 pm (the wavelength of the installed FBG is called the initial wavelength). When the track is slightly deformed due to the passing of rail vehicles, the wavelength of the FBG will move to a shorter wavelength. The wavelength offset value is negative, compared to the initial wavelength. However, the sign of wavelength shift value only represents the moving direction of wavelength, which is independent of the magnitude of track deformation. Therefore, when calculating the load, we use the unsigned value of wavelength drift.

It can be seen from Figure 9 that each FBG sensor is highly sensitive, although the response amplitude of every FBG in each experiment is not exactly the same. When the TOFC’s weight is the same, the peak difference may be due to the running speed. The wavelength shift of every sensor was more than −100 pm when the TOFC passed through. The maximum wavelength shift is −371 pm, the minimum is −155 pm, and the mean value is −225 pm.

#### 5.2.2. Force Analysis of Adjacent FBG Sensors

**Figure 10:**
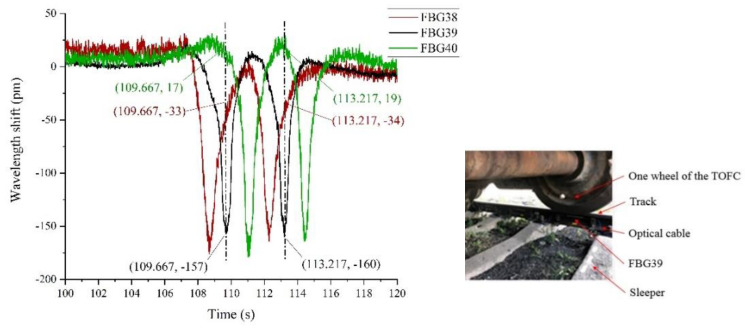
Response curves of three adjacent sensors.

We need to know how much deformation is sensed by the adjacent FBG sensors when the TOFC’s wheel passed directly through one sensor. The purpose of doing this is, firstly, to verify the correctness of the previous theoretical simulation. Secondly, if the adjacent sensors also respond to a certain extent, the installation density of the FBG sensor can be further reduced in future monitoring schemes, greatly reducing the cost of industrial projects. We selected three adjacent sensors, FBG38, 39 and 40, for analysis, and their time-domain curves are shown in Figure 10.

As can be seen from Figure 10, when the TOFC wheel passes directly above FBG39, its wavelength shift is the largest. Meanwhile, the shifts of the front and back sensors (FBG38 and FBG40) are smaller. When the front wheel passes FBG39, its maximum wavelength shifts reach −160 pm at 113.217 s, while the wavelength shifts of its neighbors FBG38 and FBG40 are 19 pm and −34 pm, respectively. When the rear wheel passes FBG39, the maximum wavelength change reaches −157 pm at 109.667 s, while the adjacent sensors, FBG38 and FBG40, have a wavelength shift of 17 pm and −33 pm, respectively. The same result can be seen at other sensing units.

This experimental phenomenon shows that in the case of a ballast track, when a train passes by, only the part of the track directly under the wheel is stressed significantly. The front and rear parts of the track are less stressed. The simulation result, that the maximum stress point is in the middle part of the track between two adjacent sleepers, is verified. Therefore, for a ballast track, if researchers need to know the force of each section of the track when a train passes by, they need to arrange for at least one sensor between every two sleepers.

#### 5.2.3. TOFC Wheel-Axle Count

For wheel-axle counting and recognition, we only need to count the number of micro-deformation signals (extremum) caused by the train; we do not need to know the actual value of the micro-strain. Judging from the previous track occupancy (Section 5.2.1), as shown in Figure 8 above, it is easy to count the number of occurrences of the maximum wavelength shift of all FBGs (or sensors beyond a certain threshold) to establish what area of track was occupied at a given moment.

**Figure 11:**
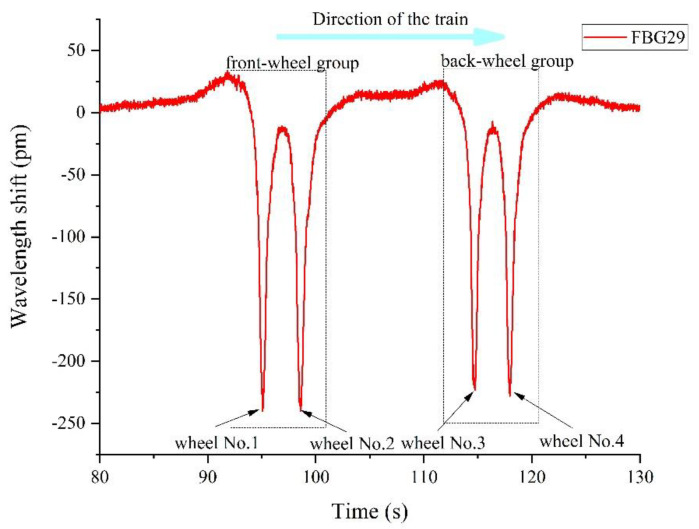
FBG29 Time domain curve.

Figure 11 shows the time domain curve of the TOFC’s wheel passing through one of the FBGs (taking FBG29 as an example). The curve of the wavelength shifts with time, when four wheels pass FBG29, can be clearly observed.

From Figure 11, we can clearly obtain the information of the front and the rear wheel groups acting on FBG29. In the Figure, there are 4 peaks where the wavelength variation of FBG29 exceeds 100 pm at different moments when the TOFC is moving. Because the FBG array is only installed on one side of the track, it can be concluded that there were 4 wheelsets on one side of the TOFC and 8 wheelsets on the whole vehicle, which is consistent with the actual situation. The sensing unit of this opto-mechatronics system is of good consistency and completed the function of wheel-axle counting.

#### 5.2.4. TOFC Length

**Figure 12:**
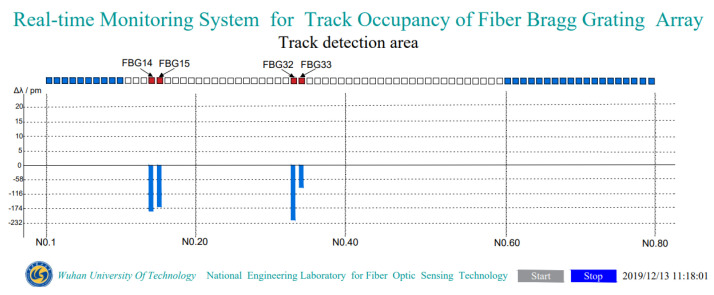
The opto-mechatronics system software interface.

The length of this TOFC can be determined by the response of the FBG array. One FBG in this track-monitoring system is installed in the middle of every two sleepers set 600 mm apart. The distance between the FBG, corresponding to the front wheel set, and the FBG corresponding to the rear wheel set is the TOFC’s length, *L_s_*:

(13) $$L_{s}=(n-1)\times s$$

where *n* is the number of sensors between two wheels of the TOFC, and *s* is the distance between two sleepers. As shown in Figure 12, the front wheelset was on FBG33 and the rear wheelset was on FBG14. There were 20 sensors between them, and the TOFC’s calculated length was 11.4 m. Compared with the actual distance between the first and fourth axles of 11.2 m, the relative error is about 1.8%.

#### 5.2.5. TOFC Moving Speed and Direction

The speed of the TOFC is determined by the response status of the array sensors and the length of the TOFC. If, at a certain moment, the wavelength shift of the *i* th FBG reaches the maximum at *t*_1_, and, after a period of time, the wavelength shift of the (*i* + 1) th sensor reaches the maximum at *t*_2_, then the speed of the TOFC can be calculated by Formula (14):

(14) $$\upsilon =\frac{L_{s}}{t_{2}-t_{1}}$$

where *L*_s_ is the distance between the front and the rear wheels of the TOFC. Taking FBG13 in Figure 8 as an example, where *t*_1_ = 76.663 s, *t*_2_ = 97.517 s, *L*_s_ = 11.4 m, the speed of the TOFC is calculated to be 0.55 m/s. According to the response of FBG31, the calculated speed is about 0.50 m/s. The calculated speed of the two points indicates that the TOFC is not running at a uniform speed. This is mainly because the flatcar is propelled by many people; it is, thus, difficult to move at a constant speed.

For the WDM/TDM networking mode, the return time of the optical signal can be obtained simultaneously when the wavelength signal of the FBG is demodulated. Through this time information, the specific distance of each FBG from the demodulation system can be obtained. The direction of the TOFC can be judged instantly, according to the meter scale value corresponding to the maximum response of the two adjacent sensors. For example, the *i* th sensor responds at a certain moment, and if the (*i* + 1) th sensor responds at the next moment, the TOFC travels in the direction from the *i* th sensor to the (*i* + 1) th sensor. If the (*i* − 1) th sensor responds at the next moment, the TOFC travels from the *i* th to the (*i* − 1) th sensor.

Based on the response of each FBG sensor, it is obvious from Figure 8 and Figure 11, above, that the TOFC moves from left to right.

#### 5.2.6. TOFC Loading

The TOFC’s weight is known to be 18 tons, with 8 wheels, that is, a single axle-bearing load of 2.25 tons. As can be seen from Figure 8 and Figure 10 above, when the TOFC wheel passed the sensor, the value of wavelength shift of the FBG was significant. According to the maximum variation of FBG wavelength caused by each axle set of the TOFC, the load of the TOFC can be calculated. Because we only installed the sensor fiber on one side of the track, the data show that the sensor had only four peaks (4 wheelsets) as the flatcar passed. Thus, the load of the TOFC should be twice the total load of the four-wheel sets. 

The weight of the TOFC can be estimated by analyzing the maximum strain of the FBG array sensors. During the running of the flatcar, a certain load is applied to the track to produce a certain stress, which can be expressed in Equation (15) [31,32]:

(15) $$\sigma =\frac{F}{S}$$

where *F* is the force exerted by the weight of the TOFC on the track, and *S* is the contact area between the wheel axle and the track. The strain value caused by stress is:

(16) $$\varepsilon =\frac{\sigma }{E}$$

where *E* = 2.06 × 10^5^ MPa is the elastic modulus of the rail, so that:

(17) F=E⋅ε⋅S

Substituting the average wavelength change of the FBG sensors, 225 pm, into Formula (3), the micro-strain of the track is 272.03 *με*. When the contact area between the wheel axle and the track is 0.4 m^2^ and the gravitational acceleration *g* = 9.8 m /s^2^, the weight of the TOFC carried by a single wheel is calculated to be 2.29 tons (relative error of 1.8%), which is closest to the actual weight.

The response of all sensors is not completely consistent, resulting in a large difference in the calculated weight of the TOFC at different times. There are several reasons for the deviation between the calculated weight value and the actual value. Firstly, to increase sensitivity, a pre-tension of 500–1000 pm was applied to each sensor in the field during sensor installation. The pre-tension of each sensor cannot be completely uniform. Secondly, the contact area of the wheelset with the track is estimated. Thirdly, the force exerted by the wheels on the sensor during the movement of the TOFC is not very accurate. As the TOFC speed increases, this value may deviate more, so it is not suitable for monitoring the weight of a train running at high speed. However, it should be more accurate during low-speed periods, such as when entering and leaving the platform. Fourthly, this method of estimating the weight of the whole flatcar according to the weight of a single wheel bearing is also one of the reasons for the weight error.

## 6. Conclusions

This paper presented an opto-mechatronics system for train-track status online monitoring, based on an FBG array with two alternate central wavelengths and low peak reflectivity (−40 dB). The optimal package and installation size of the FBG sensor were numerically calculated to ensure that the maximum wavelength shift of the FBG did not exceed 500 pm. A dual-wavelength low-reflectivity FBG array WDM/TDM hybrid multiplexing networking system, with 600 mm of spatial resolution, was used and the FBG array signal was demodulated by rule and line. The wavelength shifts of the FBG array sensors due to the movement of the experimental vehicle (TOFC) were over 100 pm (the maximum of −371 pm, the minimum of −155 pm, the mean of −225 pm). The track occupation, the TOFC’s speed (0.50–0.55 m/s) and direction, the TOFC’s load (2.29 ton), the TOFC’s length (11.4 m), and wheel axle counting (4 wheelsets on one side) can be accurately obtained in real time. This work confirms the feasibility and the potential of a low-reflectivity FBG array in long-distance, large-capacity train-track status monitoring.

## Data Availability

Data underlying the results presented in this paper are not publicly available at this time but may be obtained from the authors upon reasonable request.
